# End-Stage Renal Disease Causes Skewing in the TCR Vβ-Repertoire Primarily within CD8^+^ T Cell Subsets

**DOI:** 10.3389/fimmu.2017.01826

**Published:** 2017-12-15

**Authors:** Ling Huang, Michiel G. H. Betjes, Mariska Klepper, Anton W. Langerak, Carla C. Baan, Nicolle H. R. Litjens

**Affiliations:** ^1^Department of Internal Medicine, Erasmus MC, University Medical Center Rotterdam, Nephrology and Transplantation, Rotterdam, Netherlands; ^2^Department of Immunology, Erasmus MC, University Medical Center Rotterdam, Rotterdam, Netherlands

**Keywords:** TCR-repertoire, T cell subsets, end-stage renal disease, ageing (aging), CMV-latency

## Abstract

A broad T cell receptor (TCR-) repertoire is required for an effective immune response. TCR-repertoire diversity declines with age. End-stage renal disease (ESRD) patients have a prematurely aged T cell system which is associated with defective T cell-mediated immunity. Recently, we showed that ESRD may significantly skew the TCR Vβ-repertoire. Here, we assessed the impact of ESRD on the TCR Vβ-repertoire within different T cell subsets using a multiparameter flow-cytometry-based assay, controlling for effects of aging and CMV latency. Percentages of 24 different TCR Vβ-families were tested in circulating naive and memory T cell subsets of 10 ESRD patients and 10 age- and CMV-serostatus-matched healthy individuals (HI). The Gini-index, a parameter used in economics to describe the distribution of income, was calculated to determine the extent of skewing at the subset level taking into account frequencies of all 24 TCR Vβ-families. In addition, using HI as reference population, the differential impact of ESRD was assessed on clonal expansion at the level of an individual TCR Vβ-family. CD8^+^, but not CD4^+^, T cell differentiation was associated with higher Gini-TCR indices. Gini-TCR indices were already significantly higher for different CD8^+^ memory T cell subsets of younger ESRD patients compared to their age-matched HI. ESRD induced expansions of not one TCR Vβ-family in particular and expansions were predominantly observed within the CD8^+^ T cell compartment. All ESRD patients had expanded TCR Vβ-families within total CD8^+^ T cells and the median (IQ range) number of expanded TCR Vβ-families/patient amounted to 2 (1–4). Interestingly, ESRD also induced clonal expansions of TCR Vβ-families within naive CD8^+^ T cells as 8 out of 10 patients had expanded TCR Vβ-families. The median (IQ range) number of expanded families/patient amounted to 1 (1–1) within naive CD8^+^ T cells. In conclusion, loss of renal function skews the TCR Vβ-repertoire already in younger patients by inducing expansions of different TCR Vβ-families within the various T cell subsets, primarily affecting the CD8^+^ T cell compartment. This skewed TCR Vβ-repertoire may be associated with a less broad and diverse T cell-mediated immunity.

## Introduction

End-stage renal disease (ESRD) patients have a decreased vaccination efficacy (1–4), an increased susceptibility for infection (5–7) and a higher risk for the development of tumors (8–11). Loss of renal function is associated with a prematurely aged T cell system (12), most likely caused by the uremia-induced proinflammatory environment (13). These uremia-induced effects on T cells are expressed as a decline in thymic output, a severe depletion of naive T cell compartment, a shift to more highly differentiated memory T cell subsets, attrition of T cell telomeres (14) and a defective T cell receptor (TCR)-induced ERK phosphorylation (15).

A broad TCR-repertoire capable of recognizing a wide range of foreign antigens is crucial for adequate T cell-mediated immune responses (16). Most TCRs consist of an α and β chain and each chain is composed of a variable (V) and a constant (C) region (17). The TCR Vβ-repertoire can be assessed using several approaches such as gene scan spectratyping *via* a DNA-based PCR (18), Vβ-family phenotyping by flow-cytometry (19–21), and assessment of clonal diversity *via* next generation sequencing (NGS) (22, 23). Gene scan spectratyping of the TCR Vβ-repertoire is at best a semiquantitative measurement. Both flow-cytometry and NGS result in a more accurate quantitative assessment of the TCR Vβ-repertoire. As NGS is more labor-intensive and sorting of highly pure T cells or their subsets is required, many researchers prefer to use flow-cytometry. Flow-cytometry allows for measuring percentages of TCR Vβ-families at the T cell-subset level obviating the need for cell sorting.

We recently examined the TCR Vβ-repertoire in ESRD patients using multiplex DNA-based spectratyping. We showed ESRD to significantly and independently skew the TCR Vβ-repertoire in older individuals and this skewing was predominantly present within the CD8^+^ memory T cell compartment (24). However, details of this skewed TCR Vβ-repertoire in ESRD patients are still lacking and quantitative data related to the impact of ESRD on TCR Vβ-repertoire in the various T cell populations is rare.

During aging, the TCR Vβ-repertoire has been reported to contract (25). Aging is associated with a decline in the naive T cell compartment which possess the broadest TCR repertoire (26), and a shift toward memory T cells, developing upon encountering of an antigen and having a skewed repertoire toward particular specificities (27, 28). The prevalence of CMV-seropositivity is high amongst ESRD patients, varying from 30 to 100%, depending on socioeconomic and ethnic background (29). CMV latency profoundly affects circulating T cells resembling features of aging, including increased frequencies of more differentiated memory T cells (30, 31) and loss of telomere length (32). CMV latency may also induce contraction of the TCR Vβ-repertoire as it induces expansion of CMV-specific T cells immunocompetent donors (33) and these CMV-specific clones are stably maintained for 5 years (34). Thus, TCR Vβ-repertoire diversity may be affected by various factors.

In this study, we assessed the TCR Vβ-repertoire diversity within different T cell subsets in ESRD patients using a flow-cytometry-based taking into account the effects of aging and CMV latency.

## Materials and Methods

### Study Population

A cohort of 10 stable ESRD patients, either younger individuals (*n* = 5, age < 45 years) or older individuals (*n* = 5, age ≥ 65 years) with an oligoclonal TCR Vβ-repertoire, as determined by DNA-based spectratyping earlier (24) were studied in more detail at the T cell-subset level using a flow-cytometry based assay for TCR Vβ-repertoire analysis. Patients having a glomerular filtration rate below 15 mL/min and either or not receiving renal replacement therapy (RRT) were included. Patients were excluded from the study when having a bacterial or viral infection, malignancy, a previous transplantation or taking immunosuppressive medication (except for glucocorticoids). The patient data are compared to those generated from 10 age- and CMV-matched healthy individuals (HIs) with a polyclonal TCR Vβ-repertoire, as determined by DNA-based spectratyping (24). Lithium-heparinized blood was drawn from ESRD patients and HI. Written informed consent was obtained from all individuals included. The study was approved by the local medical ethical committee (METC number: 2012-022) and conducted according to the principles of Declaration of Helsinki and in compliance with International Conference on Harmonization/Good Clinical Practice regulations.

### Sample Preparation

Peripheral blood mononuclear cells (PBMCs) were isolated from 35 mL of lithium-heparinized blood by density centrifugation as described previously (35) and then frozen at 10 × 10^6^ PBMC per vial at −190°C until further use.

Cryopreserved PBMCs (1 vial of 10 × 10^6^ PBMCs) were thawed, counted, washed and resuspended in Isoflow™ Sheath Fluid (Beckman Coulter B.V., Woerden, Netherlands). The PBMCs were stained with Brilliant Violet 510-labeled anti-CD3 (BioLegend, Uithoorn, Netherlands), Alexa Fluor (AF)700-labeled anti-CD4 (Beckman Coulter B.V.) and Allophycocyanin (APC)-Cy7-labeled anti-CD8 (BioLegend) to identify CD4^+^ and CD8^+^ within CD3^+^ T cells. ECD-labeled anti-CD45RO (Beckman Coulter B.V.), PE-Cy7-labeled anti-CCR7 (BD, Erembodegem, Belgium), V450-labeled anti-CD31 (BD; clone WM59), peridinin chlorophyll-A protein-Cy5.5-labeled anti-CD28 (BD) and APC-labeled anti-CD57 (BioLegend) as well as fluorescence minus one controls were used to appropriately identify the different T cell subsets (illustrated in Figures S1B–D in Supplementary Material). As shown in Figure S1B in Supplementary Material, CCR7 and CD45RO are used to distinguish the different naive and memory T cell subsets, i.e., naive (CD45RO^−^CCR7^+^), central memory (CM, CD45RO^+^CCR7^+^), effector memory (EM, CD45RO^+^CCR7^−^), and terminally differentiated effector memory CD45RA^+^ T cells subsets (EMRA, CD45RO^−^CCR7^−^). CD31-expression within naive T cells (Figure S1C in Supplementary Material) identifies T cells that recently have left the thymus, also referred to as recent thymic emigrants (RTEs) (36). Loss of CD28 (CD28^−^ T cells) and gain of CD57 (CD57^+^ T cells) expression is observed in relation to increased replicative history (37, 38) and allows for identification of more differentiated T cells (Figure S1D in Supplementary Material).

Subsequently, the cell suspension was divided into eight tubes (100 μL/tube) labeled A-H, corresponding to the different antibody cocktails to stain for the 24 TCR Vβ-families (IOTest^®^ Beta Mark TCR V beta repertoire kit, Beckman Coulter B.V.). Each cocktail contains antibodies directed to three different Vβ-families, i.e., one is fluorescein isothiocyanate (FITC-), one is PE-labeled and one is labeled with both FITC and PE. Table 1 shows the description of the antibodies directed to the different TCR Vβ-families in tube A to H. A typical example of the proportions of several TCR Vβ-families within CD3^+^ T cells from tube A, tube B and tube C is depicted in Figure S1A in Supplementary Material, the last three plots.

**Table 1:** TCR Vβ-families in tube A-H.

Tube	Vβ family	Fluorochrome	Clone
A	Vβ 5.3	PE	3D11
	Vβ 3	FITC	CH92
	Vβ 7.1	PE + FITC	ZOE

B	Vβ 9	PE	FIN9
	Vβ 16	FITC	TAMAYA1.2
	Vβ 17	PE + FITC	E17.5F3

C	Vβ 18	PE	BA62.6
	Vβ 20	FITC	ELL1.4
	Vβ 5.1	PE + FITC	IMMU157

D	Vβ 13.1	PE	IMMU222
	Vβ 8	FITC	56C5.2
	Vβ 13.6	PE + FITC	JU74.3

E	Vβ 5.2	PE	36213
	Vβ 12	FITC	VER2.32
	Vβ 2	PE + FITC	MPB2D5

F	Vβ 23	PE	AF23
	Vβ 21.3	FITC	IG125
	Vβ 1	PE + FITC	BL37.2

G	Vβ 11	PE	C21
	Vβ 14	FITC	CAS1.1.3
	Vβ 22	PE + FITC	IMMU546

H	Vβ 13.2	PE	H132
	Vβ 7.2	FITC	ZIZOU4
	Vβ 4	PE + FITC	WJF24

*Detailed information with respect to the different TCR Vβ-family antibodies in tube A-H, labels and clones (IOTest^®^ Beta Mark TCR V beta repertoire kit, Beckman Coulter)*.

The samples were measured on a Navios flow cytometer (10-color configuration; Beckman Coulter B.V.) and at least 0.5 million CD3^+^ T cells were acquired for each tube. Data were analyzed by Kaluza™ software (Beckman Coulter B.V.). The number of events acquired for a specific T cell subset needed to be more than 100 to allow for reliable analysis of frequencies of TCR Vβ-families within this population. The only subset that did not meet this criterion was the EMRA population within the CD4^+^ T cells.

### Gini-TCR Index and Calculation of Expanded TCR Vβ−Families

The Gini index is used to describe the distribution of income in economic statistics. As the distribution of TCR Vβ−families shows similarities to that of income, the Gini index can also be applied in TCR Vβ-repertoire analysis by flow-cytometry. It has already been used in TCR-sequencing studies (39, 40), and was recently also introduced as an accurate and reliable way for analyzing TCR Vβ-repertoire data obtained by flow-cytometry (41). The TCR (Vβ)-Gini index with scores ranging from low to high indicates TCR Vβ-families from equal distribution (broad repertoire; i.e., low score) to unequal distribution (skewed repertoire; i.e., high score). A Microsoft excel file allowing for automatic calculation of the Gini-TCR index using percentages of 24 TCR-Vβ families is provided in the supporting file (41).

An expansion in a TCR Vβ-family in ESRD patients is defined as a frequency above the mean percentage + 2 times the SD of a certain TCR Vβ-family obtained using the HI as reference population. By using this approach, finding an expansion by chance is lower than 2.5%.

### Statistical Analyses

Gini-TCR indices or median number of expanded TCR Vβ-families/individual between two different T cell subsets within individuals were compared with Wilcoxon signed rank test and Friedman test followed by Dunn’s multiple comparison *T*-test was used for comparing more than two different T cell subsets. Trend analyses were performed using two-way ANOVA, comparing different subsets between individuals or CD4^+^ and CD8^+^ T cells. In addition, the effect of ESRD with respect to numbers of expanded TCR Vβ-families within different T cell subsets is done using Fisher’s exact test. Two-sided *P*–values <0.05 were considered statistically significant. All statistical analyses were performed with GraphPad Prism 5.

## Results

### Study Population

Detailed information of the study population is given in Table 2. Ten ESRD patients (5 younger individuals: age 20–29 years and 5 older individuals: age 65–73 years) and 10 age-matched HI (5 younger individuals age 26–42 years and 5 older individuals: age 65–73 years) were recruited into this study. Sixty percent of the ESRD and HI study population is CMV-seropositive. Seven out of 10 ESRD patients received RRT.

**Table 2:** Demographic and clinical characteristics of the study population.

	Healthy individuals	ESRD patients	*P*-value
Number of individuals	10	10	
Age (years; range)			
younger group (<45 years)	36 (26–42)	28 (20–29)	0.06
older group (>65 years)	68 (65–73)	70 (65–73)	0.92
Sex (% male)	60	40	0.66
CMV IgG serostatus (% pos)	60	60	1.00
RRT (%)	n.a.	70	n.a.
Duration of RRT (months; median/range)		20 (7–68)	
Hemodialysis (%)		85.7	
Peritoneal dialysis (%)		14.3	
Underlying kidney disease			
Primary glomerulopathy (%)		20	
Diabetic nephropathy (%)		30	
Reflux nephropathy (%)		10	
Polycystic kidney disease (%)		20	
Lupus nephritis (%)		10	
Unknown (%)		10	

*ESRD, end-stage renal disease; CMV, cytomegalovirus; pos, positive; RRT, renal replacement therapy; n.a., not applicable*.

### Gini-TCR Indices Increase with T Cell Differentiation

**Figure 1:**
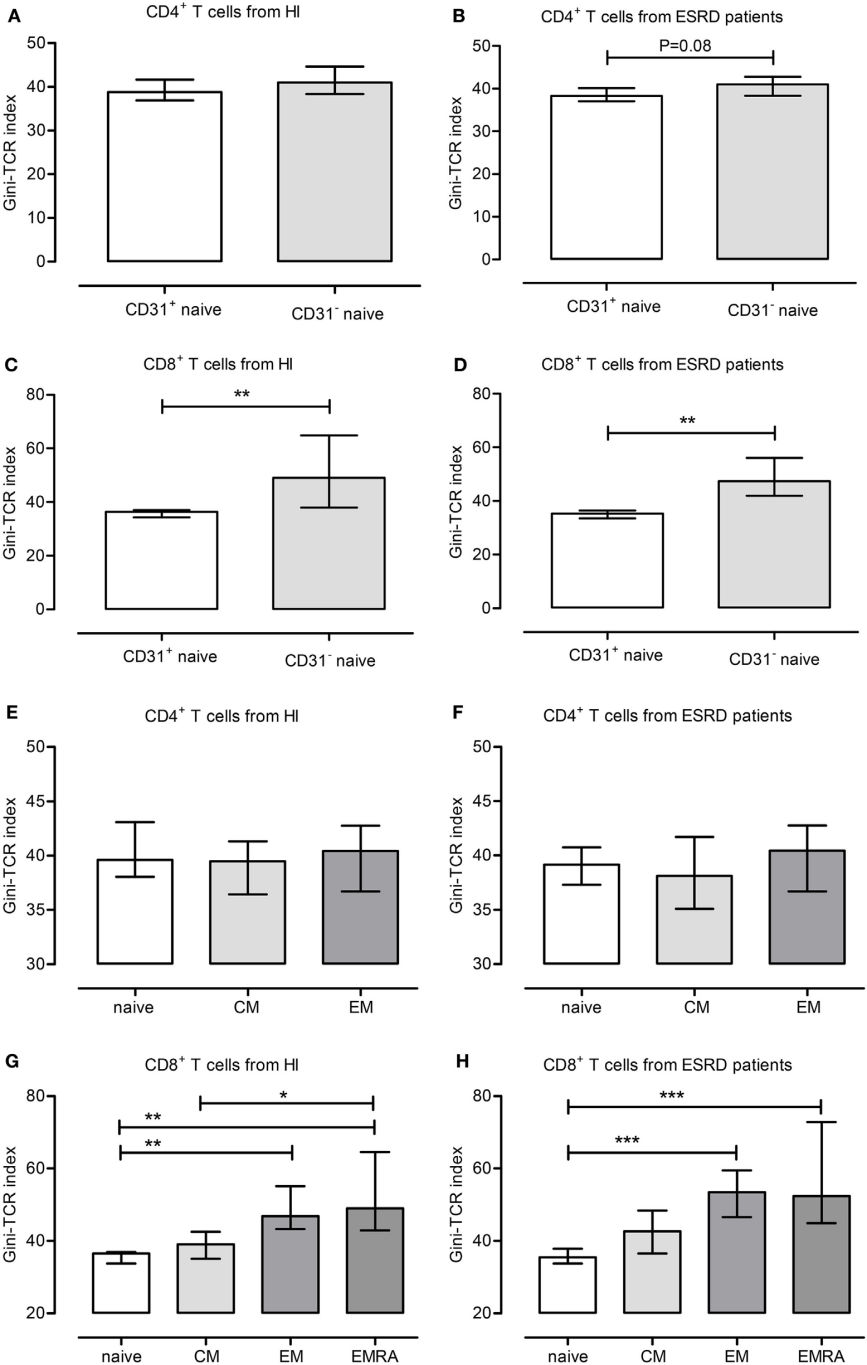
Gini-T cell receptor (TCR) indices for different T cell subsets. The different Gini-TCR indices are depicted for healthy individual (HI) **(A,C,E,G)** and end-stage renal disease (ESRD) patients **(B,D,F,H)**. First, the Gini-TCR indices for recent thymic emigrant (CD31^+^ naive) and CD31^−^ naive CD4^+^ **(A,B)** and CD8^+^ T **(C,D)** cells are given, respectively. Next, the differentiation-associated effects on Gini-TCR indices are depicted for CD4^+^ **(E,F)** and CD8^+^ **(G,H)** T cell subsets, including naive, central memory, effector memory, and terminally differentiated effector memory CD45RA^+^ (EMRA) T cells. *, **, and *** reflect *P*-values <0.05, 0.01, and 0.001, respectively. Data from 10 HI and 10 ESRD patients are given as median with interquartile range.

Naive T cells expressing CD31 are considered to be RTEs and the least-differentiated T cell subset. In ESRD patients, CD31-expressing naive T cells tended to or have a lower Gini-TCR index when compared to their CD31^−^ counterparts within CD4^+^ (Figure 1B) and CD8^+^ T cells (Figure 1D), respectively. For HI, a significant lower Gini-TCR index was only observed for CD31-expressing naive CD8^+^ (Figure 1C) but not CD4^+^ (Figure 1A) T cells when compared to CD31^−^ naive T cells. Furthermore, a T cell differentiation-associated increase in Gini-TCR indices was noted for CD8^+^ (Figures 1G,H), but not CD4^+^ (Figures 1E,F), T cells. The median value (IQ range) increased significantly (*P* < 0.001) from 36.5 (33.7–37) and 35.5 (33.8–37.9) in naive T cells to 49 (43–64.6) and 52.4 (44.9–72.8) in the highly differentiated EMRA CD8^+^ T cells for HI and ESRD patients, respectively.

### ESRD Patients Have Increased Gini-TCR Indices within Memory CD8^+^ T Cell Subsets

**Figure 2:**
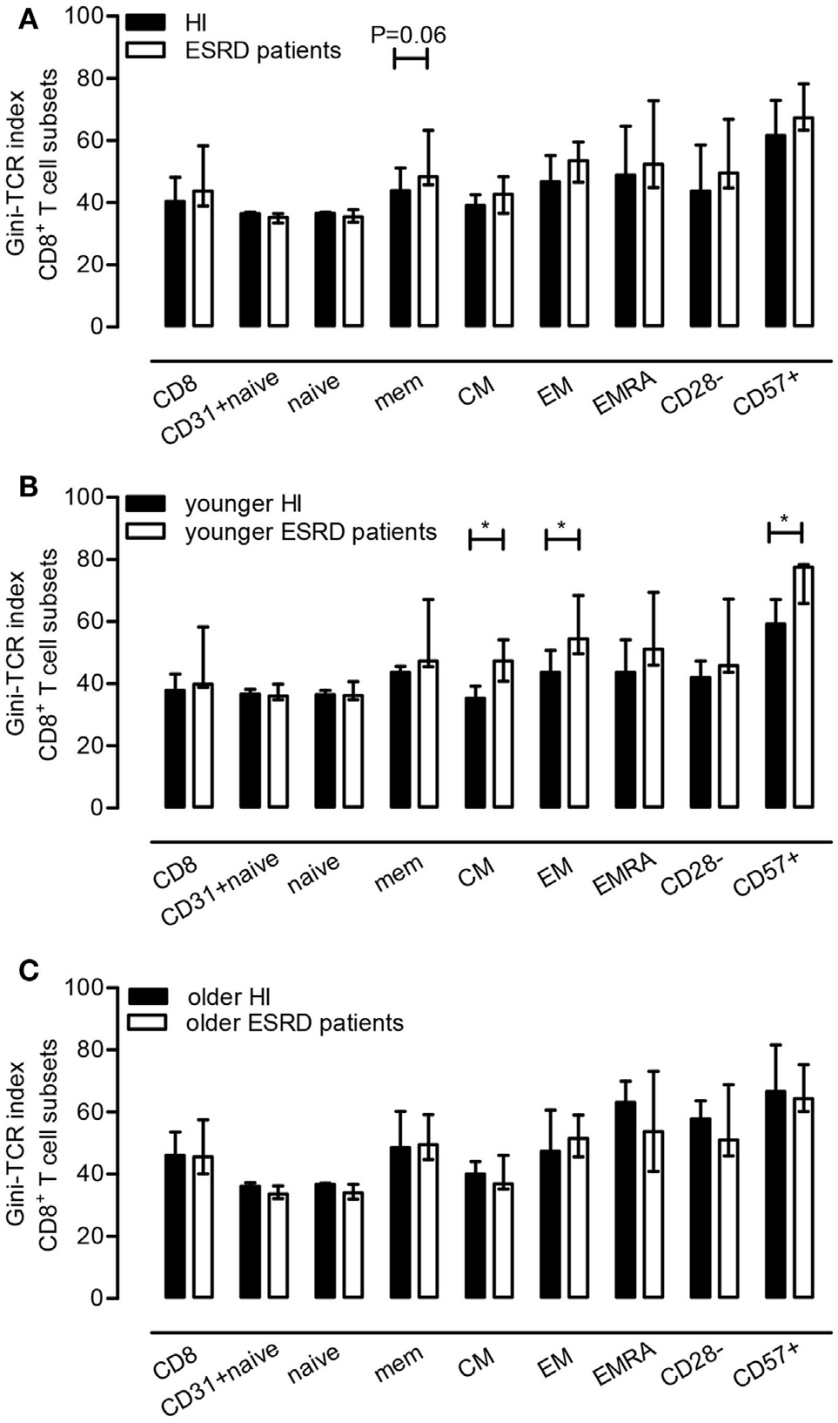
Effect of end-stage renal disease (ESRD) on Gini-T cell receptor (TCR) indices of CD8^+^ T cell subsets. In **(A)**, the median and IQ range of the Gini-TCR indices for healthy individuals (*N* = 10) and ESRD patients (*N* = 10) for different CD8^+^ T cell subsets is depicted, whereas in **(B,C)** those for the younger and older group (*N* = 5) are given, respectively. *P* value: *<0.05.

We next analyzed the influence of ESRD, aging, and CMV latency on skewing of the TCR Vβ-repertoire by comparing Gini-TCR indices for different T cell subsets including total CD3^+^ T cells, as well as naive, CD31^+^ naive, total memory (MEM), CM, EM, EMRA, CD28^−^, and CD57^+^ populations within both the CD4^+^ and CD8^+^ T cell subsets. ESRD effects with respect to Gini-TCR indices were limited to the CD8^+^ T cell compartment as it tended to induce higher Gini-TCR indices (*P* = 0.06) in CD8^+^ memory T cells when compared to HI (Figure 2A). The median (IQ range) value for Gini-TCR index in CD8^+^ memory T cells amounted to 48.4 (45.8–63.3) and 43.8 (41.1–51.2) for ESRD patients and HI, respectively. Younger (Figure 2B), but not older (Figure 2C), ESRD patients had significantly higher Gini-TCR indices within the CD8^+^ CM (*P* < 0.05), EM (*P* < 0.05) and CD57^+^ T cell compartment when compared to age-matched HI. The median (IQ range) for Gini-TCR in CD8^+^ CM, EM and CD57^+^ T cells amounted to 47.4 (40.8–54.2) versus 35.3 (34.0–39.2), 54.4 (49.6–68.5) versus 43.7 (42.4–50.8) and 77.6 (65.8–78.4) versus 59.2 (57.2–67.1) for younger ESRD patients versus younger HI.

The following results, describing Tables S1 and S2 in Supplementary Material need to be interpreted with caution as the *P*-values were not adjusted for the number of parameters compared.

Aging effects were not visible when comparing Gini-TCR indices for the different T cell subsets between younger and older ESRD patients (Table S1 in Supplementary Material). In HI, aging effects were confined to the CD8^+^ T cell compartment and an aging-related increasing trend in Gini-TCR index was observed for CD8^+^ CM T cells (*P* = 0.06), as the median (IQ range) for Gini-TCR amounted to 40.1 (39.1–44.1) in older HI versus 35.3 (34.0–39.2) in younger HI. An increased Gini-TCR index (*P* = 0.03) was observed for older HI, within CD8^+^CD28^−^ T cells (Table S1 in Supplementary Material). The median (IQ range) value for Gini-TCR in CD8^+^CD28^−^ T cells amounted to 42.0 (40.6–47.4) versus 57.8 (43.7–63.7) for younger and older HI, respectively.

CMV latency did not significantly affect Gini-TCR indices apart from a CMV-related increasing trend within CD4^+^CD57^+^ T cells (*P* = 0.07) of HI, but not ESRD patients, i.e., the median (IQ range) Gini-TCR index amounted to 67.0 (57.4–72.2) for CMV-seropositive HI versus 45.6 (35.5–58.0) for CMV-seronegative ones (Table S2 in Supplementary Material). No differences were observed when comparing CMV-seronegative and CMV-seropositive ESRD patients to their CMV-serostatus matched HI with respect to Gini-TCR indices for the different T cell subsets (data not shown).

### Clonal Expansions of TCR Vβ-Families in Different T Cell Subsets

**Figure 3:**
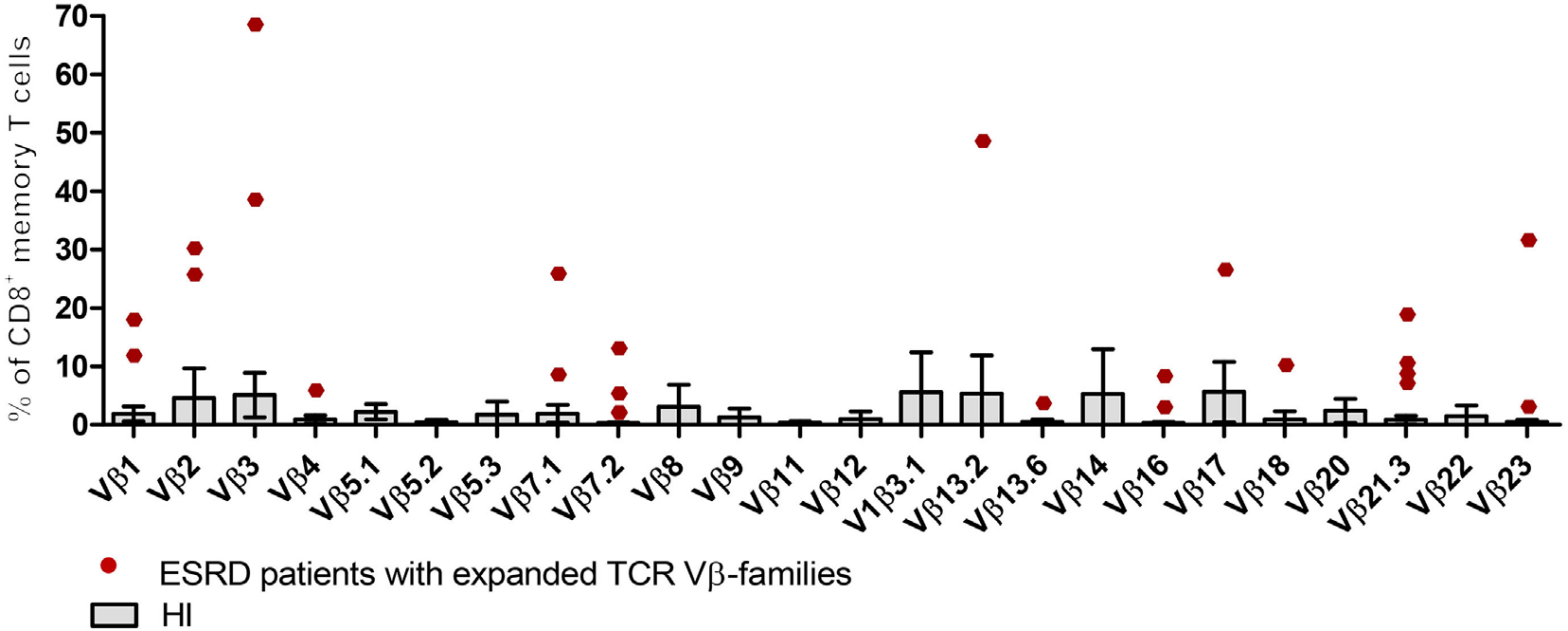
Expansions of T cell receptor (TCR) Vβ-families within CD8^+^ memory T cells from end-stage renal disease (ESRD) patients. Boxes and error bars represent the mean and 95% confidence interval (mean ± 2SD) of 24 TCR-Vβ families from 10 healthy individuals (HI). Red dots correspond to expanded TCR Vβ-families from ESRD patients (frequencies > mean + 2SD from HI).

Apart from characterizing the impact of ESRD on Gini-TCR indices for the different T cell subsets, we also evaluated the impact of ESRD on clonal expansions of TCR Vβ-families by comparing frequencies to the average + 2SD obtained using HI as reference population. Figure 3 shows a typical example of expanded TCR Vβ-families within CD8^+^ memory T cells of ESRD patients.

Clonal expansions of TCR Vβ-families were observed within the CD3^+^ T cells in 7 out of 10 ESRD patients (Table 3). Half versus all of the ESRD patients showed expanded TCR Vβ-families within CD4^+^ and CD8^+^ T cells (*P* < 0.05), respectively. The median (IQ range) number of expanded TCR Vβ-families per patient amounted to 1 (0–2) and 2 (1–4) families for CD4^+^ and CD8^+^ T cells (*P* < 0.05), respectively (Figure 4A). Interestingly, expansions were also detected within the naive T cell compartment, as 3 out of 10 and 8 out of 10 patients had expansions of TCR Vβ-families within the naive CD4^+^ and CD8^+^ T cell compartment, respectively (Table 3). Most clonal expansions were observed within the (more differentiated) memory CD8^+^ T cell subsets (Figure 4A). The median (IQ range) number of expanded TCR Vβ-families amounted to 1 (1–1) versus 2 (2–4) for naive and memory CD8^+^ T cells, respectively (*P* < 0.05). Moreover, ESRD affected different TCR Vβ-families as illustrated in Figure 3 for CD8^+^ memory T cells. ESRD induced expansions within both younger (Figure 4B) and older (Figure 4C) ESRD patients when compared to their age-matched HI.

**Table 3:** Effect of ESRD on expansions of TCR Vβ-families.

	# of ESRD patients with/without expansions	Median # of expanded TCR Vβ-families (IQ range)	Total # of expanded TCR Vβ-families
CD3^+^	7/3	1 (0–2)	15
CD4^+^	5/5	1 (0–2)	10
CD31^+^ naive	6/4	1 (0–1)	9
Naive	3/7	0 (0–1)	5
MEM	9/1	1 (1–3)	16
CM	6/4	1 (0–2)	11
EM	6/4	1 (0–2)	13
CD28^−^	9/1	1 (1–1)	12
CD57^+^	9/1	1 (0–2)	11
CD8^+^	10/0	2 (1–4)	26
CD31^+^ naive	7/3	1 (0–2)	15
Naive	8/2	1 (1–1)	12
MEM	10/0	2 (2–4)	28
CM	7/3	2 (0–3)	20
EM	9/1	4 (2–4)	32
EMRA	10/0	2 (1–3)	26
CD28^−^	10/0	3 (1–3)	28
CD57^+^	9/1	3 (1–4)	25

**Figure 4:**
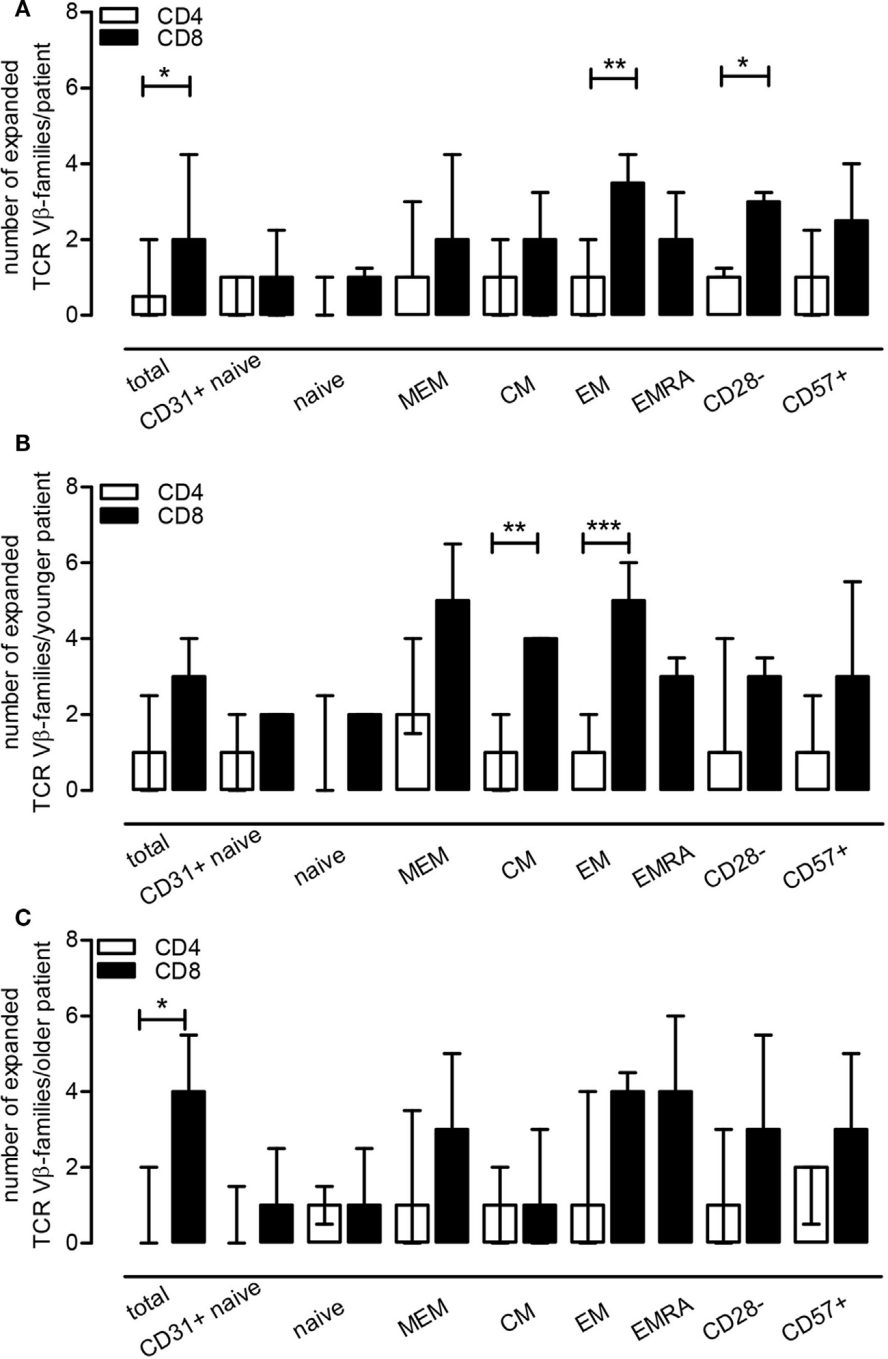
End-stage renal disease effect on T cell receptor (TCR) Vβ-families within T cell subsets. Using healthy individual (HI) (*N* = 10) as a reference population, we evaluated the number of TCR Vβ-families that were expanded per patient for a T cell subset (frequency of a TCR Vβ-family exceeding the mean + 2SD value obtained for HI) **(A)**. In **(B,C)**, the median (IQ range) of expansions per younger and older patient for a T cell subset is depicted using the younger (*N* = 5) and older HI (*N* = 5) as a reference, respectively. The open bars represent the median and IQ range for the different CD4^+^ T cell subsets, whereas the closed bars represent that for the different CD8^+^ T cell subsets.

## Discussion

The main finding of this study is that the multiparameter flow-cytometry-based approach for evaluating the skewed TCR Vβ-repertoire diversity in ESRD patients showed that TCR skewing can be observed primarily in CD8^+^ T cell subsets, including naive T cells. However, higher Gini-TCR indices, indicative for an enhanced TCR Vβ-repertoire skewing, were specifically associated with more differentiated CD8^+^, but not CD4^+^, T cell subsets in both ESRD patients and HI.

Our previous data showed that ESRD may lead to a skewed TCR Vβ-repertoire as assessed by DNA spectratyping, providing at best semiquantitative information about TCR Vβ-clonality (24). The current study provided more quantitative details with respect to this skewed TCR Vβ-repertoire at the T cell-subset level using the Gini-TCR index as a tool for calculating skewness (41) and evaluating number/type of expanded TCR Vβ-families. Higher Gini-TCR indices are indicative of a more skewed TCR Vβ-repertoire. The current study confirmed several of our previous findings. Increased skewing of the TCR Vβ-repertoire was observed for more differentiated CD8^+^, but not CD4^+^, T cells, corresponding to our spectratyping data as well as findings described by others (27, 42). In addition to the Gini-TCR index, we calculated the number of TCR Vβ-families per patient that were expanded beyond the mean + 2SD values of HI. This approach yielded similar results as the Gini-index but gives detailed information at the individual patient level for the different T cell subsets. For instance some patients have a large number of expanded TCR Vβ-families while others show only a few. Moreover, ESRD did not seem to affect one TCR Vβ-family in particular, indicative of expansions of different clonal origin. Altogether, using both Gini-TCR indices as well as the number of expanded TCR Vβ-families, revealed skewing to mainly occur within the CD8^+^ and in particular within the CD8^+^ memory T cell subset similar to what was observed before using DNA-based spectratyping on sorted T cell subsets (24).

The commercially available flow-cytometry-based assay, used to characterize the TCR Vβ-repertoire, is composed of 24 different TCR Vβ-antibodies covering about 70% of the normal human TCR Vβ-repertoire (brochure Beckman Coulter). Evaluating other TCR Vβ-families as well as TCR Vα and TCR Vγ/Vδ families may contribute to a better understanding of the whole TCR-repertoire. In this respect, γδ^+^ T cells account for approximately 8% of CD3^+^ peripheral blood T cells and around 6% of γδ^+^ T cells were observed within CD3^+^CD8^+^, but not CD4^+^ T cells in HI (19). As frequencies of γδ^+^ T cells may also vary amongst individuals, it might be more accurate to evaluate the TCR Vβ-repertoire not within total CD3^+^, like performed in the current study, but within αβ^+^ CD3^+^ T cells.

Interestingly, using this multiparameter flow-cytometry-based approach, we were also able to detect expanded TCR Vβ-families within the naive T cell compartment. This characteristic has, to our knowledge never been described for ESRD patients. Uremia induces a proinflammatory environment significantly affecting T cell-mediated immunity characterized by increased risk for infections (5) and decreased vaccination efficacy (1, 43). We have observed that progressive loss of renal function is accompanied by a severe depletion of the naive T cell compartment and a relative shift toward more differentiated memory T cells (44). Naive T cells employ a mechanism referred to as homeostatic proliferation in order to maintain the naive T cell pool that is not replenished by newly developed naive T cells from the thymus due to thymic involution. Homeostatic proliferation occurs in response to homeostatic cytokines, e.g., IL-7, or low affinity self antigens presented by antigen-presenting cells (26). This mechanism has been described to be associated with a decline in TCR Vβ-repertoire diversity within naive T cells with increasing age (27, 45). ESRD enhanced homeostatic proliferation of naive T cells to a similar extent as observed in older HI (12) and as a consequence of this compensatory mechanism, loss of TCR Vβ-repertoire diversity may also be induced by ESRD within naive CD8^+^ and/or CD4^+^ T cells.

Naive T cells are required to mount adequate immune responses to newly encountered antigens (46, 47). ESRD patients, with a severely depleted naive T cell compartment (44), are hampered in inducing adequate protection to, for example, HBV vaccination as a result of defective generation of antigen-specific memory T cells (43). The ESRD-associated defects in T cell composition as well as function, reminiscent of aging-associated T cell defects, led to the concept of premature T cell aging introduced in 2011 (12). ESRD patients have a T cell compartment that is aged by 15–20 years compared to their chronologic age, using age-matched HI as a reference. Consistent with premature T cell aging (12), we observed ESRD-associated increases in Gini-TCR indices and TCR Vβ expansions to occur already at young age.

Aging is known to affect TCR Vβ-repertoire diversity toward a more skewed pattern, starting from roughly 600 × 10^3^ clonotypes detected per 10^6^ T cells in childhood, declining by 5 × 10^3^ clonotypes per year (25). Age-related effects were limited within our cohort of HI and this may be a consequence of the selection procedure applied. We did select HI with a polyclonal (i.e., non-skewed) TCR Vβ-repertoire using DNA-based spectratyping (24), to ensure a relatively standard healthy population to be used as reference for comparison to ESRD patients. The ESRD patient population however only consisted of individuals with an oligoclonal (skewed) TCR Vβ-repertoire. This might have resulted in an underestimation of the effect of aging on TCR Vβ-families. Likewise, our selection procedure may also explain the minimal effects of CMV in both cohorts.

CMV latency is known to introduce skewing of the TCR Vβ-repertoire induced by expanded CMV-specific T cell clones in both HI (33, 34, 48) and ESRD patients (24). CMV latency may result in a vast and long-lasting expansion of CMV-specific T cells (33, 34). Moreover, CMV latency has additional effects mainly on circulating CD8^+^ T cells of ESRD patients (49).

End-stage renal disease, aging, and CMV all influenced the TCR-Vβ repertoire diversity to a different extent (24), however, because of different factors present in the environment, they all have their specific effect on clonotype selection. Even though these findings need to be verified in a larger cohort without preselection using DNA-based spectratyping of TCR-Vβ-repertoire, our study already shed some light on this altered TCR-Vβ repertoire at the T cell-subset level in particular with respect to ESRD.

Relating TCR-repertoire data to functional capacities of T cells is warranted to increase knowledge on uremia-induced T cell defects in ESRD patients. Moreover, tracking TCR clones in whole blood or tissue infiltrates may provide additional information on antigen specificity important for diagnosis of infection and allograft rejection after transplantation (50–52).

In conclusion, ESRD is associated with a skewed TCR Vβ-repertoire as a result of variable TCR Vβ-family expansions, but not one TCR Vβ-family in particular. ESRD, aging and CMV latency exert their effects by influencing different TCR Vβ-families. This altered repertoire may be associated with a less broad and less diverse T cell-mediated immunity.

## Ethics Statement

All individuals included gave informed consent and the Erasmus medical center medical ethical committee approved the study (METC number: 2012-022). It was conducted according to the principles of Declaration of Helsinki and in compliance with International Conference on Harmonization/Good Clinical Practice regulations.

## Author Contributions

LH participated in the design of the study, analyzed the data, and wrote the manuscript. MB participated in the design of the study, interpreted the data, and revised the manuscript. MK established the experimental protocol, conducted the experiments, and analyzed the data. AL participated in the design of the study, interpreted the data, and revised the manuscript. CB participated in design of the study and revised the manuscript. NL designed the study, interpreted the data, and revised the manuscript. All authors have read and approved the final manuscript.

## Conflict of Interest Statement

The authors declare that the research was conducted in the absence of any commercial or financial relationships that could be construed as a potential conflict of interest.
