# Supplementary material for: End-Stage Renal Disease Causes Skewing in the TCR Vβ-Repertoire Primarily within CD8+ T Cell Subsets
Source: Front Immunol. 2017 Dec 15;8:1826. doi: 10.3389/fimmu.2017.01826 (PMC5736542; doi:10.3389/fimmu.2017.01826)
Supplement: Supplementary file 2 [file Image_1.PDF]

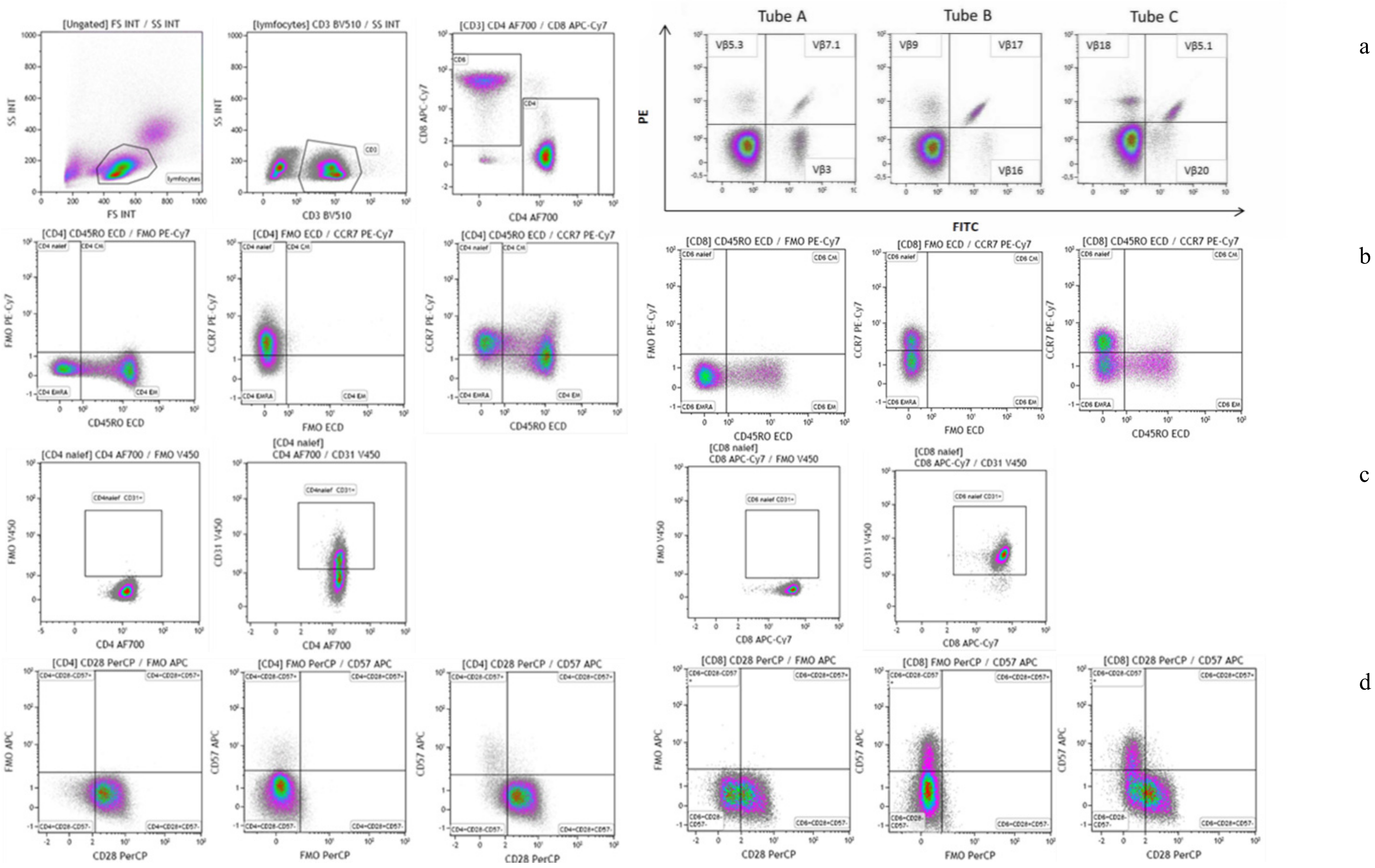

Supplementary Figure 1. Gating strategy multi-parameter flow-cytometric analysis TCR Vβ-repertoire

Briefly, lymphocytes were identified based on the forward/sideward characteristics (a, first plot) followed by the selection of $CD3^+$ T cells (a, second plot). These T cells were then dissected into $CD4^+$ and $CD8^+$ T cells (a, third plot). Furthermore, a typical flow-cytometric result for $CD3^+$ T cells with respect to tube A-C, each containing 3 different Vβ-families (identified by PE^+, PE^+FITC^+ and $FITC^+$), is depicted in the last three plots of (a). A similar approach is also followed for tubes D-H and all subsets.

Fluorescence minus one controls (FMOs) form the base for all subsequent gating performed to identify the different populations within $CD4^+$ and $CD8^+$ T cells. CCR7 and CD45RO were used to identify naïve and different memory T-cell subsets (b, first and latter 3 plots for $CD4^+$ and $CD8^+$ T cells, respectively). CD31 expression identified naïve T cells that have recently left the thymus (c, first and latter 2 plots for $CD4^+$ and $CD8^+$ T cells, respectively). Loss of CD28 and gain of CD57 expression were used for identification of highly differentiated T cells (d, first and latter 3 plots for $CD4^+$ and $CD8^+$ T cells, respectively).
