# Supplementary material for: End-Stage Renal Disease Causes Skewing in the TCR Vβ-Repertoire Primarily within CD8+ T Cell Subsets
Source: Front Immunol. 2017 Dec 15;8:1826. doi: 10.3389/fimmu.2017.01826 (PMC5736542; doi:10.3389/fimmu.2017.01826)
Supplement: Supplementary file 3 [file Table_1.docx]

|  | ESRD patients | |  | | HI | |  | |
| --- | --- | --- | --- | --- | --- | --- | --- | --- |
|  | Young (N=5) | Old (N=5) | | P-value | Young (N=5) | Old (N=5) | | P-value |
|  | median (IQ range) | median (IQ range) | |  | median (IQ range) | median (IQ range) | |  |
|  |  |  | |  |  |  | |  |
|  |  |  | |  |  |  | |  |
| CD3^+^ | 37.9 (34.6-42.8) | 35.6 (33.9-42.3) | | 0.55 | 37.1 (35.8-38.8) | 38.4 (35.5-40.1) | | 0.69 |
|  |  |  | |  |  |  | |  |
| CD4^+^ | 41.2 (37.0-42.9) | 37.9 (34.1-40.4) | | 0.25 | 41.3 (37.1-42.2) | 36.7 (35.9-42.0) | | 0.69 |
| CD31^+^ naive | 38.8 (37.5-43.6) | 37.8 (34.1-39.5) | | 0.42 | 39.8 (36.8-42.8) | 38.4 (36.7-41.8) | | 0.69 |
| naive | 40.0 (37.8-43.9) | 39.1 (34.7-40.0) | | 0.25 | 41.5 (37.9-43.7) | 38.6 (37.7-43.3) | | 0.84 |
| MEM | 41.8 (37.3-45.1) | 39.2 (36.3-40.7) | | 0.25 | 41.6 (38.2-42.5) | 40.0 (34.8-41.9) | | 0.55 |
| CM | 41.0 (36.5-44.4) | 35.3 (33.8-40.2) | | 0.15 | 39.6 (36.5-41.6) | 39.4 (35.3-41.6) | | 1.00 |
| EM | 42.5 (37.9-46.9) | 38.0 (35.7-42.1) | | 0.25 | 43.1 (37.9-43.7) | 43.2 (34.4-44.2) | | 1.00 |
| CD28^-^ | 46.3 (43.3-52.6) | 42.0 (40.0-43.8) | | 0.10 | 46.9 (43.4-56.2) | 44.5 (43.0-48.6) | | 0.55 |
| CD57^+^ | 60.3 (55.1-75.5) | 49.6 (40.5-57.4) | | 0.06 | 64.5 (45.6-73.6) | 61.9 (34.2-68.1) | | 0.55 |
|  |  |  | |  |  |  | |  |
| CD8^+^ | 39.9 (38.8-58.3) | 45.6 (40.1-57.4) | | 0.84 | 37.9 (34.2-43.1) | 46.1 (40.2-53.6) | | 0.10 |
| CD31^+^ naive | 36.1 (34.9-39.9) | 33.6 (32.2-36.2) | | 0.15 | 36.6 (34.2-38.2) | 36.2 (33.8-37.2) | | 0.84 |
| naive | 36.2 (34.8-40.7) | 33.9 (32.0-36.7) | | 0.15 | 36.4 (33.2-37.9) | 36.7 (34.1-37.1) | | 0.55 |
| MEM | 47.3 (45.5-67.1) | 49.5(44.6-59.1) | | 0.69 | 43.6 (40.3-45.6) | 48.6 (41.1-60.3) | | 0.42 |
| CM | 47.4 (40.8-54.2) | 36.9 (35.2-46.1) | | 0.15 | 35.3 (34.0-39.2) | 40.1 (39.1-44.1) | | 0.06 |
| EM | 54.4 (49.6-68.5) | 51.5 (45.6-59.0) | | 0.31 | 43.7 (42.4-50.8) | 47.3 (44.9-60.6) | | 0.31 |
| EMRA | 51.1 (45.9-69.4) | 53.8 (40.9-73.2) | | 1.00 | 43.7 (40.4-54.2) | 63.1 (46.0-70.0) | | 0.10 |
| CD28^-^ | 45.9 (43.7-67.3) | 51.0 (45.9-68.8) | | 0.55 | 42.0 (40.6-47.4) | 57.8 (43.7-63.7) | | 0.03 |
| CD57^+^ | 77.6 (65.8-78.4) | 64.3 (60.1-75.2) | | 0.31 | 59.2 (57.2-67.1) | 66.6 (54.6-81.6) | | 0.69 |
|  |  |  | |  |  |  | |  |

**Supplementary Table 1 Effect of age on Gini-TCR indices**

Median (IQ range) of Gini-TCR indices for the different T-cell subsets were compared between young (N=5) and old (N=5) using the non-parametric Mann-Whitney test. P-values<0.05 were considered statistically significant.
