# Supplementary material for: End-Stage Renal Disease Causes Skewing in the TCR Vβ-Repertoire Primarily within CD8+ T Cell Subsets
Source: Front Immunol. 2017 Dec 15;8:1826. doi: 10.3389/fimmu.2017.01826 (PMC5736542; doi:10.3389/fimmu.2017.01826)
Supplement: Supplementary file 4 [file Table_2.docx]

|  | ESRD patients | |  | | HI | | |  | |
| --- | --- | --- | --- | --- | --- | --- | --- | --- | --- |
|  | CMVneg (N=4) | CMVpos (N=6) | | P-value | CMVneg (N=4) | | CMVpos (N=6) | | P-value |
|  | median (IQ range) | median (IQ range) | |  | median (IQ range) | | median (IQ range) | |  |
|  |  |  | |  |  | |  | |  |
|  |  |  | |  |  | |  | |  |
| CD3^+^ | 35.9 (33.9-44.0) | 37.6 (34.8-41.0) | | 0.61 | 37.8 (35.8-40.6) | | 37.6 (35.6-38.6) | | 0.61 |
|  |  |  | |  |  | |  | |  |
| CD4^+^ | 38.4 (33.4-43.6) | 38.7 (37.2-41.3) | | 0.76 | 40.3 (37.0-43.8) | | 39.8 (36.1-41.4) | | 0.48 |
| CD31^+^ naive | 38.2 (33.1-45.4) | 38.3 (37.5-39.2) | | 0.91 | 41.2 (37.4-44.8) | | 38.8 (36.1-40.0) | | 0.48 |
| naive | 38.7 (33.7-45.4) | 39.2 (38.0-40.2) | | 1.00 | 41.7 (38.3-45.7) | | 39.6 (37.3-41.7) | | 0.48 |
| MEM | 39.3 (35.9-43.7) | 39.7 (38.0-42.4) | | 0.91 | 40.4 (37.2-43.1) | | 40.2 (36.3-42.0) | | 0.76 |
| CM | 37.8 (33.4-42.8) | 38.1 (35.7-42.2) | | 0.61 | 39.0 (36.6-42.8) | | 39.5 (35.6-40.4) | | 0.61 |
| EM | 39.6 (35.1-43.1) | 40.4 (37.6-44.6) | | 0.76 | 40.7 (36.1-44.9) | | 43.1 (37.2-43.3) | | 0.76 |
| CD28^-^ | 43.7 (39.6-49.8) | 43.8 (41.3-48.3) | | 0.91 | 45.4 (43.3-46.7) | | 47.0 (43.4-53.7) | | 0.26 |
| CD57^+^ | 47.7 (39.1-56.6) | 62.1 (50.6-75.3) | | 0.11 | 45.6 (35.5-58.0) | | 67.0 (57.4-72.2) | | 0.07 |
|  |  |  | |  |  | |  | |  |
| CD8^+^ | 40.5 (38.5-54.2) | 49.8 (39.5-58.9) | | 0.48 | 40.4 (35.4-44.9) | | 42.9 (37.1-53.3) | | 0.61 |
| CD31^+^ naive | 34.6 (31.7-41.7) | 35.4 (34.1-36.4) | | 0.91 | 35.6 (34.1-37.4) | | 36.5 (34.1-37.5) | | 0.91 |
| naive | 34.7 (31.4-41.8) | 35.5 (34.0-37.9) | | 0.76 | 35.4 (33.4-36.8) | | 36.7 (33.7-37.7) | | 0.48 |
| MEM | 46.9 (43.8-61.6) | 52.8 (46.3-63.6) | | 0.48 | 43.0 (41.3-47.4) | | 45.5 (40.5-59.6) | | 0.76 |
| CM | 47.6 (38.1-49.6) | 40.0 (36.5-47.7) | | 0.61 | 40.7 (34.9-42.7) | | 37.6 (35.1-41.4) | | 0.91 |
| EM | 55.9 (47.7-60.2) | 53.0 (46.4-63.2) | | 0.76 | 46.9 (43.7-52.0) | | 45.8 (43.1-60.1) | | 0.91 |
| EMRA | 46.3 (38.8-66.7) | 59.7 (49.4-73.0) | | 0.35 | 48.3 (42.1-60.2) | | 51.8 (42.7-69.4) | | 0.76 |
| CD28^-^ | 45.9 (42.6-63.3) | 58.6 (45.7-67.6) | | 0.35 | 43.0 (40.0-54.4) | | 47.6 (42.7-62.3) | | 0.48 |
| CD57^+^ | 67.4 (62.0-75.3) | 71.9 (63.1-79.5) | | 0.76 | 57.7 (52.6-64.7) | | 67.1 (58.1-81.2) | | 0.17 |
|  |  |  |  | |  |  | | |  |

**Supplementary table 2 Effect of CMV on Gini-TCR indices**

Median (IQ range) of Gini-TCR indices for the different T-cell subsets were compared between CMV-seronegative (CMVneg; N=4)

and CMV-seropositive (CMVpos; N=6) using the non-parametric Mann-Whitney test.

P-values<0.05 were considered statistically significant.
